# Screening for *Candida auris* in patients admitted to eight intensive care units in England, 2017 to 2018

**DOI:** 10.2807/1560-7917.ES.2021.26.8.1900730

**Published:** 2021-02-25

**Authors:** Ashley Sharp, Berit Muller-Pebody, Andre Charlett, Bharat Patel, Rebecca Gorton, Jonathan Lambourne, Martina Cummins, Adela Alcolea-Medina, Mark Wilks, Robin Smith, Damien Mack, Susan Hopkins, Andrew Dodgson, Phillipa Burns, Nelun Perera, Felicia Lim, Gopal Rao, Priya Khanna, Elizabeth Johnson, Andrew Borman, Silke Schelenz, Rebecca Guy, Joanna Conneely, Rohini J Manuel, Colin S Brown

**Affiliations:** 1 Field Epidemiology Training Programme, Public Health England, London, United Kingdom; 2 National Infection Service, Public Health England, London, United Kingdom; 3 Health Service Laboratories, LLP, London, United Kingdom; 4 Barts Health NHS Trust, London, United Kingdom; 5 Department of Infection, Royal Free London NHS Foundation Trust, London, United Kingdom; 6 Manchester University NHS Foundation Trust, Manchester, United Kingdom; 7 University Hospitals of Leicester NHS Trust, Leicester, United Kingdom; 8 London North West University Healthcare NHS Trust, London, United Kingdom; 9 Royal Brompton Hospital, London, United Kingdom

**Keywords:** Candida auris, intensive care, hospital-associated infection, antimicrobial resistance, emerging infection

## Abstract

**Background:**

*Candida auris* is an emerging multidrug-resistant fungal pathogen associated with bloodstream, wound and other infections, especially in critically ill patients. *C. auris* carriage is persistent and is difficult to eradicate from the hospital environment.

**Aim:**

We aimed to pilot admission screening for *C. auris* in intensive care units (ICUs) in England to estimate prevalence in the ICU population and to inform public health guidance.

**Methods:**

Between May 2017 and April 2018, we screened admissions to eight adult ICUs in hospitals with no previous cases of *C. auris,* in three major cities. Swabs were taken from the nose, throat, axilla, groin, perineum, rectum and catheter urine, then cultured and identified using matrix-assisted laser desorption/ionisation time-of-flight mass spectrometry (MALDI-TOF MS). Patient records were linked to routine ICU data to describe and compare the demographic and health indicators of the screened cohort with a national cohort of ICU patients admitted between 2016 and 2017.

**Results:**

All *C. auris* screens for 921 adults from 998 admissions were negative. The upper confidence limit of the pooled prevalence across all sites was 0.4%. Comparison of the screened cohort with the national cohort showed it was broadly similar to the national cohort with respect to demographics and co-morbidities.

**Conclusion:**

These findings imply that *C. auris* colonisation among patients admitted to ICUs in England is currently rare. We would not currently recommend widespread screening for *C. auris* in ICUs in England. Hospitals should continue to screen high-risk individuals based on local risk assessment.

## Introduction

*Candida auris* is an emerging fungal pathogen first described in Japan in 2009 [1]. It is associated with bloodstream, wound and other infections, especially in critically ill patients [2,3]. *C. auris* usually has intrinsic resistance to fluconazole and has the propensity for other resistance, with multidrug-resistant isolates described [4]. Prolonged hospital outbreaks have been described and have been regarded as difficult to control despite extensive infection control measures [5]. Cases of *C. auris* have been reported on five continents, with outbreaks reported in India, Pakistan, South Africa and Venezuela [4]. Laboratory identification of *C. auris* has been challenging, requiring up-to-date reference databases for matrix-assisted laser desorption/ionisation time-of-flight mass spectrometry (MALDI-TOF MS), or use of genotypic methods [6,7]. A 2018 update of the European Centre for Disease Prevention and Control (ECDC) rapid risk assessment of *C. auris* in healthcare settings [7] noted a substantial increase in the number of reported cases in European countries, with Spain and the United Kingdom (UK) most affected. In addition, a survey by the ECDC in 2020 reported variable levels of laboratory capacity and overall preparedness for *C. auris* screening [8].

In England, 225 cases of *C. auris* were reported between June 2013 and March 2017 (164 colonisations and 61 infections including 31 candidaemias) across 22 hospitals, and three substantial outbreaks were reported in intensive care units (ICUs) in London and Oxford [5,9,10]. No deaths were directly attributable to *C. auris.* Surveillance for *C. auris* in England comprises: routine reporting into the national laboratory reporting system Second Generation Surveillance System (SGSS) from laboratories with the capacity to detect *C. auris*, routine reporting from the national mycology reference laboratory (MRL) which takes referrals from laboratories unable to identify *C. auris,* and reports of cases and clusters from local health protection teams (HPTs) [11]. *C. auris* is not on the list of statutory notifiable causative organisms. The English surveillance programme for antimicrobial utilisation and resistance (ESPAUR) performed a mycology laboratory capacity survey in 2017 which showed that only 53% of responding laboratories (n = 47) could discriminate *C. auris* from other *Candida* species locally, with others referring suspicious isolates to the MRL for species confirmation or identification [12].

Admission of patients with asymptomatic colonisation may be an important source of infection in hospital outbreaks, especially in ICUs where there is evidence of high transmissibility and persistence [5,9], along with increased likelihood of progression to invasive disease. Admission screening can be a helpful tool to prevent hospital transmission by enabling rapid infection control measures. Screening also provides epidemiological data that can inform public health policy, for example by identifying risk factors for colonisation, which can inform further targeting of screening practice. National guidance for methicillin-resistant *Staphylococcus aureus* (MRSA) [13] recommends screening of all patients admitted to high-risk units. In addition, some units screen for other resistant organisms such as carbapenemase-producing Enterobacterales (CPE), according to local epidemiology [14]. Contemporaneous national guidelines [6] for *C. auris* recommended that all hospitals develop a screening policy after local risk assessment for those patients most likely to be colonised. The policy should include screening of contacts of cases, previously colonised individuals, and patients coming from affected hospitals in the UK and abroad. Patients admitted to ICU with a history of hospitalisation in a country with a high prevalence of drug-resistant pathogens would ideally be routinely placed in contact precautions in a private room [15], but this may not be possible due to bed capacity. Importantly, *C. auris* cases have been reported in the UK with no history of travel or exposure to an affected hospital (data not shown), suggesting there may be non-negligible endemicity in the UK. *C. auris* could be another candidate for routine admission screening in high-risk settings if a cost-effectiveness analysis demonstrated its utility. This would depend on the attributed burden of disease, the availability of effective control measures to prevent transmission, the prevalence of colonisation and the number needed to screen to find positive cases.

We aimed to pilot admission screening for *C. auris* in ICUs in England, in order to estimate the prevalence of colonisation in the ICU population and to inform public health guidance.

## Methods

### Study setting and design

Eight adult ICUs in England were purposively selected for inclusion in this study. The ICUs had to be able to rapidly incorporate the screening protocol into routine practice without additional staffing. The hospitals had to have no known cases or outbreaks of *C. auris,* so we could be confident that cases were genuine introductions, rather than acquired in the hospital itself prior to ICU admission. We excluded hospitals that shared patient populations with hospitals with ongoing outbreaks. We hypothesised that communities with high rates of travel to and from other countries affected by *C. auris* would have a higher prevalence, and thus be a potential target for screening. So we selected hospitals that serve ethnically diverse communities, expected to have a high rate of travel, based on Office for National Statistics figures (11.8 million trips in 2018) [16]. Ethnicity itself was not a factor of interest, rather intended to serve as a crude proxy for travel.

Participating hospitals were located in three major cities in England: London (6), Leicester (1) and Manchester (1). We offered screening to all patients admitted to participating ICUs during the screening period regardless of source of admission (emergency department, operating theatre, current inpatient in the hospital or directly transferred from another hospital). Hospital selection was not random so formal sample size calculation was not possible, but we calculated an indicative sample size assuming a prevalence of 0.5%. With 1,000 screens, we would expect to achieve a precision of ± 0.5% assuming a design effect of 1.3. We asked hospitals to start screening as soon as possible and continue until a target of 150–200 screened admissions was reached. Due to the varying set-up times across hospitals, screening start dates were staggered over 9 months. The duration of screening in each hospital also varied depending on size of the ICU and capacity to screen. The overall study ran for 12 months between May 2017 and April 2018.

### Screening method and laboratory diagnostic

The culture-based screening method was chosen by expert consensus. We collected samples within 24 hours of admission to ICU at the same time as other routine screening samples. We collected swabs from: (i) nose; (ii) throat; (iii) axilla; (iv) groin; (v) perineum; (vi) rectum; and (vii) a catheter urine sample. Swabs were transported to the clinical laboratory in standard tubes without viral transport media or any solution with antifungal activity. All except one laboratory were on the participating hospital sites. Laboratory diagnosis was undertaken based on an agreed standard operating procedure. Swabs and urine were directly streaked onto Sabouraud Dextrose agar plates (SDA) (or CHROMagar followed by sub-culture on SDA) and incubated aerobically at 37 °C for 7 days, checking for yeast growth every 24 hours. Morphologically distinct colonies were sub-cultured before identification or were directly identified using MALDI-TOF MS. Six of the seven laboratories (one laboratory served two ICUs) used the Bruker MALDI-TOF MS system (Bruker, Karlsruhe, Germany) and one used the VITEK MALDI-TOF MS system (bioMerieux, Craponne, France). We collected data on the laboratory results for each body site tested, along with patient identifiers.

### Demographic and health information and comparison cohort

Demographic and health information for the screened cohort was obtained by linking deterministically to data from the national Case Mix Programme (CMP) database based on NHS number, hospital number, date of birth, post code and sample/admission date. The CMP is the national clinical audit of patient outcomes from adult critical care coordinated by the Intensive Care National Audit and Research Centre (ICNARC). We described the cohort with respect to demographic and health indicators and compared them to a national cohort of 170,540 admissions from all adult general critical care units (n = 216) in England, Wales and Northern Ireland between 1 April 2016 and 31 March 2017, using t-tests, chi-squared tests and Wilcoxon rank-sum tests as appropriate. We considered a p value of < 0.05 to be statistically significant. Clopper-Pearson exact binomial 95% confidence intervals were applied to provide interval estimates of overall and ICU-specific prevalence.

### Ethical statement

This protocol was approved by the Public Health England (PHE) Research Ethics and Governance Group.

## Results

Table 1 shows the screening activity by hospital unit for the eight ICUs involved in the study. In total, 921 adults were screened over 998 admissions (some patients were admitted to ICU multiple times) between May 2017 and April 2018. Those ICUs that reached the target of around 150–200 screened admissions ceased screening before the end of the study. Of the 998 admissions screened, the proportion of each body site tested was: nose (850/998, 85%), throat (534/998, 54%), axilla (819/998, 82%), groin (638/998, 64%), perineum (773/998, 77%), rectum (638/998, 64%), and urine (684/998, 69%). Three ICUs did not report any throat swabs, one ICU did not any report rectal swabs and one ICU did not report any groin swabs.

**Table 1:** *Candida auris* screening activity by hospital and body site tested, England, 2017–2018 (n = 998)

Hospital ICU	Start month	End month	Total days screened	Admissions screened	Nose		Throat		Axilla		Groin		Perineum		Rectum		Urine	
			n	n	n	%	n	%	n	%	n	%	n	%	n	%	n	%
Hospital A	May 2017	July 2017	55	154	142	92	142	92	146	95	141	92	137	89	137	89	124	81
Hospital B	June 2017	Mar 2018	284	97	90	93	0	0	90	93	80	82	80	82	80	82	46	47
Hospital C	July 2017	Sep 2017	65	76	58	76	54	71	25	33	10	13	18	24	58	76	46	61
Hospital D	July 2017	Sep 2017	64	169	133	79	133	79	135	80	28	17	134	79	129	76	112	66
Hospital E	Aug 2017	Apr 2018	267	98	76	78	0	0	76	78	72	73	72	73	72	73	55	56
Hospital F	Oct 2017	Jan 2018	92	168	143	85	0	0	143	85	135	80	135	80	135	80	116	69
Hospital G	Dec 2017	Mar 2018	81	191	180	94	177	93	177	93	172	90	169	88	0	0	163	85
Hospital H	Jan 2018	Feb 2018	23	45	28	62	28	62	27	60	0	0	28	62	27	60	22	49
**Total**	**NA**	**NA**	**NA**	**998**	**850**	**85**	**534**	**54**	**819**	**82**	**638**	**64**	**773**	**77**	**638**	**64**	**684**	**69**

ICU: intensive care unit; NA: not applicable.

Records collected for this study were successfully linked to records in the CMP database for 95.7% (881/921) of patients and 90.9% (907/998) of admissions. Of the 40 patients that could not be linked to the database, 26 failed because ICNARC had not yet received/validated the routine ICU data and 14 failed due to missing or incomplete identifiers. These were excluded from the case mix analysis (Tables 2 and 3), but were included in the screening activity (Table 1) and screening results (Table 4). Table 2 shows patient characteristics for the 881 linked patients (based on data from the first admission), compared to the national cohort. The screened cohort was similar to the national cohort with respect to age and sex. The ethnic mix of the screened cohort was significantly different (p < 0.001) to the national cohort. However, the proportion of those screened that were non-UK residents was similar in both cohorts. The proportion of screened patients with severe conditions recorded in their medical history was similar to the national cohort. However, the proportion of screened patients with end-stage renal failure, haematological malignancy or a compromised immune system was slightly higher than in the national cohort (p < 0.001).

**Table 2:** Characteristics of patients screened for *Candida auris*, and national cohort patients, with medical records linked to the Case Mix Programme database, England, 2017–2018

Patient characteristics	Screened cohort^a^ n = 881		National cohort^b,c^ n = 162,695		p value
	n	%	n	%	
Age (years; mean, SD)	59.0	17.7	60.9	18.0	0.001^d^
**Sex**					
Female	357	40.5	73,050	44.9	0.009
Male	524	59.5	89,645	55.1	
**Ethnicity**					
White	561	63.7	143,047	87.9	< 0.001^e^
Mixed	4	0.5	975	0.6	
Asian	145	16.5	6,335	3.9	
Black	80	9.1	4,120	2.5	
Other	49	5.6	2,409	1.5	
Not stated	42	4.8	5,809	3.6	
**Residency**					
Non-UK resident^f^	3	0.3	681	0.4	0.74^e^
**Severe conditions in medical history^g^**					
Severe liver disease	26	3.0	4,413	2.7	0.66^e^
Very severe cardiovascular disease	25	2.9	2,715	1.7	0.007^e^
Severe respiratory disease	32	3.7	3,860	2.4	0.014^e^
End-stage renal failure	56	6.4	3,559	2.2	< 0.001^e^
Haematological malignancy	34	3.9	3,244	2.0	< 0.001^e^
Metastatic disease	25	2.9	5,740	3.5	0.27^e^
Immunocompromised	99	11.3	12,767	7.9	< 0.001^e^
**Prior dependency**					
Able to live without assistance	623	71.1	123,133	76.0	< 0.001^e^
Some minor/major assistance	249	28.4	36,976	22.8	
Total assistance with all daily activities	4	0.5	1,813	1.1	

SD: standard deviation; UK: United Kingdom.

^a^ 881/921 screened patients had records that were successfully linked to the Case Mix Programme database.

^b^ These data derive from the Case Mix Programme database. The Case Mix Programme is the national clinical audit of patient outcomes from adult critical care coordinated by the Intensive Care National Audit and Research Centre (ICNARC). For more information on the representativeness and quality of these data, please contact ICNARC (https://www.icnarc.org/).

^c^ All patients admitted to adult general critical care units between 1 April 2016 and 31 March 2017.

^d^ t-test.

^e^ Chi-squared test.

^f^ Based on 865 patients with a UK postcode or country of residence recorded.

^g^ Based on 876 patients with evidence available to assess past medical history.

**Table 3:** Admission characteristics for patients screened for *Candida auris* with medical records linked to the Case Mix Programme database and for national cohort patients, England, 2017–2018

Admission characteristics	Screened cohort^a^ n = 907		National cohort^b^ n = 170,540		p value
**Source of admission**	**n**	**%**	**n**	**%**
Emergency department, unplanned	297	32.7	45,575	26.7	< 0.001^c^
Emergency department, planned	7	0.8	819	0.5	
Operating theatre following elective surgery, planned	94	10.4	33,807	19.8	
Operating theatre following elective surgery, unplanned	15	1.7	6,363	3.7	
Operating theatre following emergency surgery	176	19.4	31,666	18.6	
Ward or intermediate care area	248	27.3	44,354	26.0	
Other critical care unit, repatriation	14	1.5	1,175	0.7	
Other critical care unit, planned or unplanned	51	5.6	5,403	3.2	
Other acute hospital not critical care	5	0.6	1,378	0.8	
**Severity scores**	**Mean**	**SD**	**Mean**	**SD**	**p value**
ICNARC physiology score	17.8	8.8	16.4	9.0	< 0.001^d^
APACHE II Acute Physiology Score^e^	11.8	5.6	11.1	5.9	< 0.001^d^
APACHE II Score^e^	16.2	6.8	15.4	6.8	< 0.001^d^
**ICNARC_H-2015_ model**	**Median**	**IQR**	**Median**	**IQR**	**p value**
ICNARC_H-2015_ model predicted risk of acute hospital mortality	8.4	2.2–30.7	7.4	1.9–26.9	0.014^f^

APACHE II: Acute Physiology And Chronic Health Evaluation II; ICNARC: Intensive Care National Audit and Research Centre; IQR: interquartile range; SD: standard deviation.

^a^ 907/998 screened patients had records that were successfully linked to the Case Mix Programme database.

^b^ These data derive from the Case Mix Programme database. The Case Mix Programme is the national clinical audit of patient outcomes from adult critical care coordinated by the Intensive Care National Audit and Research Centre (ICNARC). For more information on the representativeness and quality of these data, please contact ICNARC (https://www.icnarc.org/).

^c^ Chi-squared test.

^d^ t-test.

^e^ Excluding admissions aged less than 16 years and admissions staying less than 8 hours in the critical care unit.

^f^ Wilcoxon rank-sum test.

**Table 4:** *Candida auris* screening results by hospital intensive care unit, England, 2017–2018 (n = 921)

Hospital ICU	Patients screened (n)	Positive (n)	Positivity (%)	Lower 95% CI^a^	Upper 95% CI^a^
Hospital A	154	0	0	0	2.4
Hospital B	96	0	0	0	3.8
Hospital C	58	0	0	0	6.2
Hospital D	143	0	0	0	2.5
Hospital E	96	0	0	0	3.8
Hospital F	154	0	0	0	2.4
Hospital G	177	0	0	0	2.1
Hospital H	44	0	0	0	8.0
**Total**	**921**	**0**	**0**	**0**	**0.4**

CI: confidence interval; ICU: intensive care unit.

^a^ Clopper-Pearson exact binomial 95% CI.

Table 3 details the admission characteristics for the 907 linked admissions of screened patients, compared with the national cohort of patients admitted to adult general critical care units participating in the CMP between April 2016 and March 2017. The source of screened admissions differed from the national cohort (p < 0.001), with a higher proportion of admissions coming from the emergency department or operating theatre following emergency surgery and a lower proportion of admissions coming from the operating theatre following elective surgery. The severity of illness on admission was slightly higher for the screened cohort, with a mean ICNARC physiology score of 17.8 vs 16.4 (p < 0.001) and a median predicted risk of mortality of 8.4 vs 7.4 (p < 0.001), respectively.

All *C. auris* screens were negative. A pooled estimate across all intensive care unit sites provided an upper limit for the Clopper-Pearson exact binomial 95% confidence interval of 0.4% (Table 4).

## Discussion

We screened 998 admissions (921 individuals) to eight ICUs in three major cities in England using a novel multiple body-site screening methodology. All *C. auris* screens were negative. We compared the screened cohort with a national cohort and showed they were broadly similar with respect to demographics (except ethnicity) and co-morbidities. A non-random sampling strategy was used to select participating hospitals, so it is not possible to directly infer prevalence. Confidence intervals are presented but these are indicative only. The upper confidence limit of the pooled prevalence across all sites was 0.4%.

A key limitation of this study is the non-random sampling method and limited geographical representativeness, with all participating hospitals residing in three major cities. A true prevalence estimate would require a nationally representative survey, such as the 2016 ECDC point prevalence survey of healthcare-associated infections (HAI) and antimicrobial use in European acute care hospitals [17], which included 32 hospitals and 20,148 patients in England. A point prevalence survey was not feasible given the time and budget constraints of our study. Screening patients admitted over a period of time allows for greater numbers per hospital and fewer participating hospitals, although this approach obscures any changes in prevalence during the period. We do not know if the overall prevalence changed during the study period, but we can say that there were no new outbreaks of *C. auris* observed, and routine surveillance only identified 30 cases during this time, including nine infections and 21 colonisations, with four imported and 26 linked to existing outbreaks or cases (data not shown). Routine surveillance data show no evidence of a seasonal trend in candidaemia [18], so we have no reason to expect seasonality in *C. auris* infection, though again this has yet to be established. At the time of the present study, there was considerable uncertainty about the epidemiology of this emerging pathogen, and an urgent need for evidence, so feasibility and speed were important factors in protocol development. The prolonged set-up time and variation in protocol reflect the challenges of incorporating a new screening programme into routine practice with a limited budget and no dedicated additional staffing.

We selected hospitals that had not previously been affected by *C. auris.* Many patients admitted to ICU have already spent time in hospital, whether in another ward, another critical care unit, or in the operating theatre, and there is evidence that *C. auris* can persist in ward environments [5,9]. Had we included hospitals with outbreaks of *C. auris* we would not have known whether cases were colonised before admission to hospital, or after admission to hospital but before admission to ICU. By focussing on hospitals with no known transmission we could be confident that any cases detected were imported from the community. We could have included hospitals with small numbers of sporadic cases to increase the likelihood of finding a case on admission, but such sporadic cases were generally associated with international travel (data not shown), so may not reflect the local epidemiology. Some admission screening had already taken place in the three hospitals that reported substantial outbreaks between 2013–­17, but unfortunately those data were not available for inclusion in this study. The other factor influencing hospital selection was that hospitals serving ethnically diverse communities were expected to have a high rate of travel back and forth to other affected countries [2,3] and thus likely to have higher prevalence of *C. auris* and be higher priority for screening. With no cases identified and with no other geographical areas to compare with we were not able to draw any conclusions about the difference in prevalence between communities.

We used routinely collected ICU data to describe our screened cohort with respect to health and demographic indicators and to enable within-cohort comparison and comparison to the national cohort. As no *C. auris* cases were detected, it was not possible to examine factors associated with colonisation, such as source of admission or co-morbidities. We could only compare the screened cohort with the national cohort to give an indication of representativeness. The national cohort statistics that we compared with were taken from a year earlier, but national cohort indicators are relatively stable over time [19] so the comparison is valid. Overall, we found that the screened cohort was broadly similar to the national cohort with respect to demographics (except ethnicity) and co-morbidities. It is important to note that small differences (e.g. mean age 59.0 vs 60.9 years, p = 0.001) are more likely to reach statistical significance due to the large size of the national cohort (170,540 admissions from 162,695 patients). We noted a significant difference in ethnicity, as was expected due to the selection of hospitals described above. The source of admission for the screened cohort differed somewhat from the national cohort, with more emergency admissions and fewer elective surgical admissions, which presumably explains the slight difference in severity of illness.

Culture of *Candida* species on SDA is well established and successful culture of *C. auris* has been demonstrated on SDA and CHROMagar [20]. Identification of yeasts was performed using the Bruker MALDI-TOF MS system, which has been shown in previous studies to be successful in the identification of *C. auris* [3,21]. Only one hospital (Hospital A) used a different system, the Vitek MALDI-TOF MS system, which did not have the updated database to be able to correctly identify *C. auris.* However, the hospital had access to a reference laboratory to test for suspicious isolates. In a separate study, we sent isolates of *C. auris* to this reference laboratory and had them tested using their Vitek MALDI-TOF MS. All tests resulted in ‘no identification’ and none were misidentified as a different species [22]. In the present study, all the screening samples that grew *Candida* species were successfully identified to species level, with no cases of ‘no identification’. Therefore, we can be confident that *C. auris* was not missed.

The culture-based screening method was chosen by expert consensus. The sensitivity and specificity of this screening method for *C. auris* detection has not been previously described. It is possible that the sensitivity of this method may be lower than would be desirable for a screening programme. Patients generally carry commensal yeasts in small numbers until their normal flora is disrupted by exposure to antimicrobials or they become immunosuppressed. Colonisation with *Candida* has been linked to duration of stay in an ICU facility [23]. Thus, the sensitivity of a screening culture for *C. auris* may be dependent on severity of illness, length of ICU stay, or exposure to antimicrobials. It may be possible to increase the sensitivity by adding an enrichment broth step, such as a salt yeast nitrogen base dulcitol/mannitol broth, which has been demonstrated to isolate *C. auris* from clinical and environmental specimens [24]. However, it was not possible to do this in the present study due to the prohibitive additional time and cost required, as well as challenges of implementation into routine laboratory practice. Furthermore, the sensitivity and specificity of screening could have been enhanced by using PCR-based detection rather than culture [25]. This was not possible at the time of the study due to time and cost restrictions, though it is an important method to consider for future *C. auris* screening strategies. It may be possible to increase apparent sensitivity by sampling patients after 7–10 days in the ICU after which colonisation with yeast and yeast infections generally becomes more prevalent [23,26,27]. The focus of this study was the admission prevalence of *C. auris,* and delayed or repeat screening was not performed as it would have been difficult to interpret, however, this will be the focus of future work.

The choice of body sites to screen was based on previous publications suggesting colonisation of these sites was common in cohorts exposed to *C. auris* [5]. The differences in body sites screened at certain hospitals were due to local differences in routine practice and operational challenges. This may have adversely affected sensitivity, though all hospitals screened at axilla sites and all but one screened groin, which are the most common sites of colonisation [28]. Our findings cannot support or refute the inclusion of a given body site in future screening. We do not know the effect of the application of chlorhexidine body wash on *C. auris*, which is widely used in the UK on admission to hospital.

This screening was incorporated into routine practice at an estimated cost of GBP 15–30 per screen. To screen all 170,540 ICU admissions in England, Wales and Northern Ireland in 2016–17 would have cost GBP 2.5–5 million. Given the low prevalence and current low burden of disease known to be attributable to *C. auris*, widespread screening would unlikely be cost effective, despite the large costs associated with outbreaks described above. However, there remains a risk of introduction to high-risk settings and further outbreaks with potentially high cost and morbidity. Current guidelines recommend all hospitals develop a screening policy after local risk assessment [6]. Screening is recommended in ICUs that have ongoing cases and/or colonisations, or on identification of a new infected or colonised patient, and admission screening is advised for patients coming from other affected hospitals/ICUs in the UK and abroad. Current guidelines also reinforce the need to analyse to species level any *Candida* species isolates associated with invasive infection, as well as any isolates from superficial sites in patients from high intensity/augmented care settings or who have been transferred from a *C. auris* affected hospital [6].

### Conclusion

We screened a large number of patients on admission to ICUs in England and we found no cases of *C. auris* colonisation. We can be somewhat reassured from this that the prevalence of *C. auris* among patients admitted to ICUs in England is currently low. Widespread screening in ICUs in England is unlikely to be cost-effective and is not currently recommended. Hospitals should continue to screen high-risk individuals on admission based on local guidance, and wider admission screening should be considered based on local risk assessment. Repeated screening or the use of molecular detection methods may be important to increase the sensitivity of the screening method.
